# Factors Influencing eHealth Literacy among Spanish Primary Healthcare Users: Cross-Sectional Study

**DOI:** 10.3390/ijerph192315497

**Published:** 2022-11-23

**Authors:** David García-García, María Julia Ajejas Bazán, Francisco Javier Pérez-Rivas

**Affiliations:** 1 Nursing Primary Health Care Service of Madrid, 28004 Madrid, Spain; 2 Academia Central de la Defensa, Escuela Militar de Sanidad, Ministerio de Defensa, 28040 Madrid, Spain; 3 Grupo de Investigación UCM “Salud Pública-Estilos de Vida, Metodología Enfermera y Cuidados en el Entorno Comunitario”, Departamento de Enfermería, Facultad de Enfermería, Fisioterapia y Podología, Universidad Complutense de Madrid, 28040 Madrid, Spain; 4 Red de Investigación en Cronicidad, Atención Primaria y Promoción de la Salud—RICAPPS—(RICORS), Instituto de Investigación Sanitaria Hospital 12 de Octubre (imas12), 28041 Madrid, Spain

**Keywords:** eHealth literacy, primary care nursing, primary healthcare, public health, sociodemographic factors

## Abstract

Background: Adequate eHealth literacy levels empower people to make informed decisions, enhancing their autonomy. The current study assessed a group using primary care services for their eHealth literacy and examined its relationship with sociodemographic characteristics. Methods: Adult patients in need of primary care nursing services participated in this cross-sectional study, which was carried out in a healthcare center in the Madrid region of Spain. Through systematic random sampling, 166 participants were chosen for the study. The eHealth Literacy Questionnaire was used to assess eHealth literacy (eHLQ). Results: The studied population showed higher eHealth literacy scores in dimensions 2 (“understanding of health concepts and language”) and 4 (“feel safe and in control”); the lowest scores were recorded for dimensions 1 (“using technology to process health information”), 3 (“ability to actively engage with digital services”), and 7 (“digital services that suit individual needs”). People with completed secondary education and a better-perceived health status who were younger and employed showed a higher level of eHealth literacy. Conclusions: The findings advance our knowledge of the variables affecting eHealth literacy. We may be able to understand patients’ needs and provide them with greater support if we can pinpoint the areas where they demonstrate the lowest eHealth literacy.

## 1. Introduction

The number of people who access and receive information through the internet has grown progressively. Due to this digitalization, websites have become the primary supplier of health-related data. It is estimated that approximately half of patients search for information related to their symptoms prior to seeking professional advice [1]. It is also believed that six out of ten Europeans use the internet to resolve doubts related to health problems, and nine out of ten trust the information they find [2]. This information may be wrong or inaccurate and, if not critically evaluated, can lead to the adoption of behaviors that can potentially harm our health [1,3]. eHealth can be defined as “the efficient and secure use of the information and communication technologies (ICT) for health support and its related fields” [4]. Interventions such as telehealth, telemedicine, mobile health (m-health), big data, or electronic medical records can be included in it [4]. The utilization of and interaction with eHealth are known as eHealth literacy, which is defined as “the ability to search, find, comprehend and evaluate health information through electronic sources and apply the knowledge acquired to tackle and solve a health-related issue” [3].

Adequate eHealth literacy levels can result in electronic health information being beneficial, since it empowers people to make informed decisions, enhance their autonomy, and promote the adoption of a healthy lifestyle. In addition, good eHealth literacy is directly related to improved health outcomes, healthcare cost reduction, increased motivation to seek health information, better knowledge about chronic diseases, the adoption of preventive and positive health behaviors, and a better self-perception and concern for one’s own health [1,3].

Currently, the scales that are most commonly used are the “eHealth Literacy Scale (eHEALS)”, an 8-item questionnaire that collects information on knowledge, safety in use, and perceived skills in eHealth [5], and the “eHealth Literacy Questionnaire (eHLQ)”, a 35-item questionnaire designed to provide multidimensional information on eHealth literacy that helps in understanding and evaluating the interaction of people with digital health services. The eHLQ was chosen for this study, as it gathers data on eHealth literacy as a whole, in contrast to other tools, offering a comprehensive grasp of its definition. Additionally, it serves a constructivist purpose: the data gathered can be used to identify the steps necessary to raise eHealth literacy [6]. Seven domains are evaluated by the 35 items in the questionnaire: using technology to process health information (dimension 1), understanding of health concepts and language (dimension 2), ability to actively engage with digital services (dimension 3), feel safe and in control (dimension 4), motivated to engage with digital services (dimension 5), access to digital services that work (dimension 6), and digital services that suit individual needs (dimension 7), each of which is a reliable, independent indicator of eHealth literacy. The following scale is used to grade these statements: strongly disagree, disagree, agree, and highly agree (scored as 1–4, in that order). The questionnaire does not provide a total score but gives an independent score for each dimension. The results also shed light on the caliber of eHealth services offered because the questionnaires gather information on people’s experiences using digital technologies to acquire, evaluate, and employ health knowledge, manage health, and communicate with healthcare practitioners. Sociodemographic characteristics, such as age, sex, and socioeconomic and educational levels, can condition eHealth literacy levels. The few studies available in the literature that have evaluated the impacts of these factors describe how years of schooling and higher socioeconomic status increase eHealth literacy levels. Age is frequently linked to lower eHealth literacy levels, and regarding gender, there are no conclusive data [3]. There are also correlations between eHealth literacy and how well people manage their own health [2]. Additionally, cultural variations might result in disparities in values and perspectives that could affect eHealth literacy and the availability of healthcare services [7]. Overall, eHealth inequities can be mainly modified with general and health education and the quality, safety, and accessibility of health services and health policies [1,3,7,8,9].

The current study´s objectives were to assess the eHealth literacy of a population using primary care services in Madrid (Spain) and to investigate the association between this literacy and sociodemographic variables.

## 2. Materials and Methods

### 2.1. Study Design and Participants

The STROBE principles for observational research were followed in the design of this cross-sectional study [10]. Patients using primary care nursing services at a healthcare facility in the Madrid region (Spain) made up the study population. All interested parties provided informed consent (provided in the Spanish language) in order to be included, and candidates had to be older than eighteen years of age and inclined to engage willingly. Subjects with language challenges (non-Spanish speakers) that prevented them from understanding the study or the questionnaire/forms utilized therein were also omitted, as were those who were going through an acute procedure that would make them uncomfortable or limit their ability to participate. People who needed immediate care (such as those with fever, headaches, general pain, etc.) and those who had major mental illnesses or cognitive decline were not included.

Based on data from a prior study on health literacy, the sample size was calculated, assuming the population to be infinite [11]. The present research included 166 subjects. For patients scheduled to receive care from four nurses, systematic probabilistic sampling was carried out to ensure that the sample is a faithful representation of the population that attends the services of the Primary Care Health Center. This population belongs to a neighborhood with an area of 361 hectares containing 57,390 inhabitants, with 159 inhabitants/hectare. The population is aged and belongs predominantly to the middle class. The first subject chosen corresponded to the first subject in the schedule, the second subject corresponded to the fourth, and the third subject corresponded to the seventh.

### 2.2. Procedures

The data-gathering technique was taught to the four previously stated nurses. The trial was fully explained to the patients, and all of their questions were answered. The study’s voluntary and charitable nature was stressed. The subjects were given the research details, the consent form, the sheet for collecting sociodemographic characteristics, and the eHLQ. Once the questionnaires were completed, the information was entered into an Excel document by the principal investigator.

Each patient’s level of support needed to complete the questionnaire was evaluated. Those who did not need assistance were moved to a different room so they could finish it independently of the nurse’s presence. The questionnaire was self-administered in an effort to eliminate (as far as feasible) any “social desirability” bias. Questionnaires that had been completed correctly were returned and reviewed. To clear up any ambiguities, null or nonsensical answers were evaluated and finished alongside the subject. When a high level of assistance was thought to be required (for example, because of poor vision, illiteracy, etc.), investigators filled out the questionnaire alongside the subject using an interview style. Individuals were scheduled to return on a different day at an appropriate time if they required more assistance or did not have enough time to finish the questionnaire.

### 2.3. Outcome Measures

The primary variable, i.e., the degree of eHealth literacy, was measured using the eHLQ (measured as a quantitative, discrete variable). The eHLQ was created using a validity-driven methodology, giving it good validity and reliability, and has been validated to date for the English, Spanish, German, and Mandarin languages [6,12,13]. The Spanish eHLQ was employed in this investigation.

Data on sociodemographic factors included sex, age, education, country of birth, marital status, occupation, and perceived health status. The cut-off point utilized for education was secondary education. This methodology was chosen since it would provide greater differences between groups when transformed into a dichotomous variable (categories: incomplete secondary education and completed secondary education or higher) due to the literacy levels between them being higher. This also facilitates the comparison of the results with other eHealth literacy studies, as this cut-off point is the most utilized in the literature.

### 2.4. Statistical Analysis

The eHealth literacy dimension scores were found to be regularly distributed using the Kolmogorov–Smirnov test. To determine whether there were differences in the mean scores of the sociodemographic variables, Student’s *t*-test was used (for the statistical treatment, the variables were recoded as dichotomous variables). The *p* value was < 0.05. The effect was calculated as Cohen’s d for mean differences (with 95% confidence intervals). For values of >0.20–0.50, the effect size was deemed minor; for 0.50–0.80, it was deemed medium; and for >0.80, it was deemed substantial. Additionally, forward stepwise multiple linear regression was carried out to verify the impact of sociodemographic factors on the results for the various dimensions. The statistical software IBM SPSS Statistics 27TM was used for all calculations.

The mean values were calculated from the scores of the questions evaluated following the instructions for using the eHLQ. Greater eHealth literacy is reflected in a higher score. It should be noted that the domain scores from the eHLQ do not yield an overall score for eHealth literacy [6,14].

### 2.5. Ethics Approval

The project was accepted by the Ethics Committee of “Gerencia Asistencial de Atención Primaria de Madrid” (protocol code 01/22-c approved on 24 January 2022) and by “Universidad Complutense de Madrid” (protocol code CE_20220120-10_SAL approved on 20 January 2022).

## 3. Results

### 3.1. Description of the Sample

The total number of participants in the study was 166, of which 45.2% were men and 54.8% were women. The median age was 65 years with a range of 73 (between 21 and 94 years) and an interquartile range of 26 (between 52 and 78). In regard to education, 8.4% were illiterate or had incomplete primary studies, 31.3% had primary education and 9.6% had secondary education, 25.3% completed high school or had professional training, and 25.3% had a university education. Of those surveyed, 91% were Spanish, with the remaining 9% being of foreign origin. In relation to marital status, 22.3% were single, 53% were married, 6.6% were separated, and 18.1% were widowed. In terms of employment status, 32.5% were employees, 4.2% were self-employed, 2.4% were unemployed, 50.6% were retired or pensioners, 7.2% did unpaid domestic work, 1.8% were students, and 1.2% were not classifiable. Finally, the perceived state of health was very bad in 0.6% of the cases, bad in 3%, regular in 35.5%, good in 53.6%, and very good in 7.2%.

### 3.2. eHealth Literacy Levels

**Figure 1:**
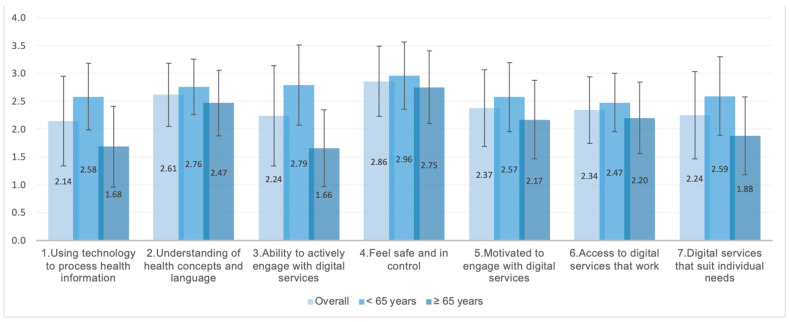
eHLQ dimension scores for each age group (under 65 and over 65) and for the entire study population. Mean and standard deviation for responses to the eHealth Literacy Questionnaire (eHLQ). Scores for each dimension ranged from 1 to 4. Higher values equal better eHealth literacy. All differences by age are statistically significant.

The highest eHealth literacy values for the entire study population were obtained for dimensions 2 (“understanding of health concepts and language”) and 4 (“feel safe and in control”), and the lowest values were for dimensions 1 (“using technology to process health information”), 3 (“ability to actively engage with digital services”), and 7 (“digital services that suit individual needs”) (Figure 1). The age group under 65 scored significantly higher across all dimensions relative to those over 65.

### 3.3. eHealth Literacy Levels according to the Sociodemographic Characteristics

The statistically significant differences observed were that those under 65 years old scored higher than those over 65 years in every dimension. Male subjects obtained higher scores than female subjects in dimension 5, “motivated to engage with digital services”. Employed subjects scored higher in every dimension when compared with the unemployed group. With the exception of dimension 4 (“feel safe and in control”), individuals with completed secondary education scored higher compared to individuals with incomplete secondary education. Subjects who perceived their health to be good or very good scored higher in every dimension compared to those who perceived their health to be fair, poor, or very poor, with the exception of dimension 2, “understanding of health concepts and language”. Those born outside the country scored higher than Spanish individuals in dimension 1, “using technology to process health information”. Married people scored higher when compared with single, separated, or widowed individuals in dimensions 4 (“feel safe and in control”) and 6 (“access to digital services that work”). The largest effect sizes (ESs) were observed for dimension 1, “using technology to process health information”, with respect to age group (ES = −1.26 [−1.61, −0.94]) and education (ES = 1.28 [0.95, 1.64]) and for dimension 3, “ability to actively engage with digital services”, with respect to age group (ES = −1.47 [−1.83, −1.14]), occupation (ES = −1.25 [−1.61, −0.92]), and education (ES = 1.29 [0.96, 1.65]) (Table 1).

### 3.4. Predictive Factors of the Level of eHealth Literacy

The scores for dimensions 1 (“using technology to process health information”), 3 (“ability to actively engage with digital services”), 6 (“access to digital services that work”), and 7 (“digital services that suit individual needs”) were found to be positively correlated with being under 65 years old. The variable “sex” was solely included in the model for dimension 7, “digital services that suit individual needs”; men obtained higher values. Being employed was a positive predictor for dimension 4, “feel safe and in control”. Dimensions 1 (“using technology to process health information”), 2 (“understanding of health concepts and language”), 3 (“ability to actively engage with digital services”), 5 (“motivated to engage with digital services”), and 7 (“digital services that suit individual needs”) were negatively affected by having incomplete secondary education. Having a health status that was considered to be very bad, bad, or fair had an adverse effect on dimensions 1 (“using technology to process health information”), 5 (“motivated to engage with digital services”), 6 (“access to digital services that work”), and 7 (“digital services that suit individual needs”). None of the dimensions were affected by the birth country. Dimensions 1 (“using technology to process health information”), 4 (“feel safe and in control”), 5 (“motivated to engage with digital services”), and 6 (“access to digital services that work”) were all negatively correlated with being single, separated, or widowed (Table 2).

## 4. Discussion

The results of this study show that the population who attended the Primary Care Health Center faced a number of difficulties. This might have been influenced by the medium-low socioeconomic level of the subjects, as digital health literacy is closely related to the socioeconomic level [3,7,15,16].

The highest scores were obtained for dimensions 2 (“understanding of health concepts and language”) and 4 (“feel safe and in control”), and the lowest were for dimensions 1 (“using technology to process health information”), 3 (“ability to actively engage with digital services”), and 7 (“digital services that suit individual needs”). Interestingly, the subjects scored higher in areas that are not directly associated with the utilization of digital services, whilst the lowest scores were obtained for dimensions that depend mostly on that interaction. This may explain the low eHealth literacy scores in all dimensions, indicating that the population does not actively use technology for health purposes. In two of the studies that were found to utilize the eHLQ, performed by Cheng et al. [12,17], the same dimensions were reported as the highest ones, with the lowest being very similar. The scores were higher in all dimensions except for dimension 4, “feel safe and in control”, when compared with the present study, which could indicate that Cheng´s study population was more accustomed to the use of digital services. This could be attributed to the high ownership of digital devices by the participants

When the dimensions were subdivided by age category, the younger population had significantly higher scores in all of them, and young age was positioned as a positive predictor for dimensions 1 (“using technology to process health information”), 3 (“ability to actively engage with digital services”), 6 (“access to digital services that work”), and 7 (“digital services that suit individual needs”). This might indicate that the younger population is more accustomed to using technology since they are “technological natives”, which exhibits a great difference based on age, in accordance with other studies [3,7,15,18]. These results are in agreement with the integrative review by Herrera et al. [15], which states that older age is associated with the decreased adoption and usage of healthcare technologies and holds the most prejudice against them. Accordingly, the analysis of Makowsky et al. [19] (who used the eHEALS) revealed that people over 65 had considerably lower eHLQ scores, and age was negatively correlated with the levels of eHealth literacy. A study that utilized the eHLQ [12] had the same conclusions, with age having an overall negative effect on all of its dimensions.

Sex generated differences with respect to dimension 5, “motivated to engage with digital services”, with the male population returning higher scores, and was a positive predictor for dimension 7, “digital services that suit individual needs”. This might have been influenced by the fact that, traditionally, men were more likely to choose technology-related professions in comparison with women. Although the same differences were found in the study by Cheng et al. [12], the literature is inconclusive [3], and some studies position the female sex as a protective factor [18,20]. The study of Tran et al. [21] (who used the eHEALS) reported higher scores in male participants, which might not be representative due to the sample being taken from nursing students and the proportion of men being only about a 6.9% of the total sample.

Employed individuals scored higher than unemployed ones for all parameters. The former was additionally established as a positive predictor for dimension 4, “feel safe and in control”. No prior research on eHealth literacy has investigated variations by employment status. However, the fact that, in modern society, the majority of jobs require the development of digital skills might explain these findings. It is important to mention that these results may be conditioned by the level of education, since people with a higher education level may have higher employment rates.

As expected, the educational level played a key role in determining digital health literacy levels. The population with completed secondary education had statistically significantly higher eHLQ scores, with the exception of dimension 4, “feel safe and in control”. This is considered to be understandable, as having more education does not necessarily translate into feeling safer. In addition, incomplete secondary education negatively affected the majority of dimensions. These results are consistent with the existing literature [2,15,19,22]. The study of Cheng et al. [12] reported that education had a positive impact on all eHLQ dimensions except for dimensions 4 (“feel safe and in control” (as in the present study)) and 6 (“access to digital services that work”). Similarly, two studies conducted in nursing students [23,24], the subjects being young and well educated, had high eHLQ scores.

Health literacy and perceived health status were found to have a strong positive association. Those who perceived their health status as good or very good scored higher in every dimension, with the exception of dimension 2, “understanding of health concepts and language”. This might indicate that patients who consider their health status to be not as good are actually the ones that are most vulnerable when interacting with digital services, which can have an even worse effect on their health status. In line with the most recent literature, health status is a crucial determining element in how well healthcare technology is adopted, since a more compromised health status may make its use difficult [15].

Comparing Spanish-born subjects to foreign subjects produced variations in relation to dimension 1, “using technology to process health information”, with the latter group returning better scores. This might indicate that Spanish people are not as used to utilizing digital health services as people from other countries. However, it is crucial to take into consideration that the study population only contained a very small proportion of individuals who were born abroad, with those being young and well educated; this limits the generalization of any interpretations, in addition to this variable not being included as a predictor in any of the analyses. The study of Cheng et al. [12] reported a negative impact of speaking a foreign language at home on the eHLQ level in all of its dimensions, which cannot be compared with the present study, as the foreigners included were from Spanish-speaking countries.

Those who were single, separated, and widowed scored significantly lower for dimensions 1 (“using technology to process health information”), 4 (“feel safe and in control”), 5 (“motivated to engage with digital services”), and 6 (“access to digital services that work”), indicating that people who are “alone” might face challenges in these areas. Only one study that analyzed this was found [22] (in which the eHEALS was used), with the results showing that single people had lower eHealth literacy than people who were married. Nevertheless, these results might not be comparable to those of the present study, as the sample was limited to people between 40 and 64 years old.

Due to its cross-sectional design, the current study has a drawback: while associations can be found, causality cannot be established. Additionally, because the study was carried out in a single facility that served a population with a moderately poor socioeconomic status, the findings may solely apply to health domains with similar characteristics. Due to the tiny available subsample, caution should be taken when interpreting the data for people who were born abroad. However, this study used a careful sampling strategy, which significantly improves the internal consistency of the results. Additionally, a large cohort was possible since patient eligibility criteria were not very limiting, improving the results’ generalizability. In addition, this is one of the very few articles to date that has analyzed the influence of sociodemographic characteristics on the eHealth literacy of a population that attends the services of a Primary Care Health Center, reducing the lack of information in this regard.

Future studies should examine the connection between the social environment and eHealth literacy, as well as the possible effects of health education initiatives intended to increase eHealth literacy and accessibility to digital services offered to healthcare consumers.

## 5. Conclusions

The current study adds to our knowledge of the variables influencing eHealth literacy and identifies patient vulnerability hotspots. Healthcare practitioners and other stakeholders may make use of this knowledge to achieve adequate levels of eHealth literacy in order to lessen health disparities, encourage the sustainable utilization of health services, and encourage users of the healthcare system.

## Figures and Tables

**Table 1:** Associations among sociodemographic factors and eHLQ dimensions.

Variable	1. Using Technology to Process Health Information	2. Understanding of Health Concepts and Language	3. Ability to Actively Engage with Digital Services	4. Feel Safe and in Control	5. Motivated to Engage with Digital Services	6. Access to Digital Services That Work	7. Digital Services That Suit Individual Needs
**Age group**							
<65 years							
Mean (SD)	**2.58 (0.62)**	**2.75 (0.51)**	**2.78 (0.73)**	**2.96 (0.61)**	**2.57 (0.62)**	**2.48 (0.53)**	**2.59 (0.71)**
n = 81							
≥65 years							
Mean (SD)	**1.72 (0.73)**	**2.49 (0.59)**	**1.71 (0.72)**	**2.76 (0.65)**	**2.19 (0.71)**	**2.21 (0.63)**	**1.91 (0.71)**
n = 85							
*t*-test (F; *p*)	6.523; **0.000**	2.767; **0.003**	0.541; **0.000**	0.065; **0.038**	2.161; **0.000**	0.722; **0.004**	0.014; **0.000**
Effect Size (95% CI)	−1.26 (−1.61, −0.94)	−0.47 (−0.79, −0.16)	−1.47 (−1.83, −1.14)	−0.32 (−0.63, −0.01)	−0.57 (−0.89, −0.26)	−0.46 (−0.78, −0.16)	−0.95 (−1.29, −0.64)
**Sex**							
Male							
Mean (SD)	2.20 (0.79)	2.58 (0.51)	2.35 (0.91)	2.84 (0.58)	**2.50 (0.66)**	2.40 (0.56)	2.37 (0.78)
n = 75							
Female							
Mean (SD)	2.09 (0.81)	2.64 (0.61)	2.15 (0.89)	2.87 (0.68)	**2.28 (0.71)**	2.29 (0.62)	2.14 (0.78)
n = 91							
*t*-test (F; *p*)	0.033; 0.379	0.361; 0.519	0.060; 0.157	0.495; 0.759	0.555; **0.040**	0.567; 0.219	0.000; 0.059
Effect Size (95% CI)	−0.14 (−0.45, 0.17)	0.11 (−0.20, 0.41)	−0.22 (−0.53, 0.08)	0.05 (−0.26, 0.36)	−0.32 (−0.63, −0.01)	−0.18 (−0.50, 0.12)	−0.29 (−0.61, 0.01)
**Occupation**							
Employed							
Mean (SD)	**2.61 (0.59)**	**2.77 (0.48)**	**2.84 (0.71)**	**3.02 (0.59)**	**2.61 (0.62)**	**2.50 (0.53)**	**2.64 (0.70)**
n = 61							
Unemployed							
Mean (SD)	**1.86 (0.78)**	**2.52 (0.59)**	**1.87 (0.81)**	**2.76 (0.64)**	**2.24 (0.70)**	**2.24 (0.62)**	**2.01 (0.73)**
n = 103							
*t*-test (F; *p*)	13.998; **0.000**	3.226; **0.005**	4.816; **0.000**	0.033; **0.010**	2.959; **0.001**	1.030; **0.006**	0.116; **0.000**
Effect Size (95% CI)	−1.04 (−1.40, −0.72)	−0.45 (−0.78, −0.13)	−1.25 (−1.61, −0.92)	−0.42 (−0.74, −0.10)	−0.55 (−0.88, −0.23)	−0.44 (−0.77, −0.12)	−0.87 (−1.22, −0.55)
**Education**							
Incomplete secondary education							
Mean (SD)	**1.62 (0.69)**	**2.45 (0.61)**	**1.64 (0.77)**	2.75 (0.71)	**2.08 (0.68)**	**2.17 (0.69)**	**1.85 (0.74)**
n = 66							
Completed secondary education or higher							
Mean (SD)	**2.49 (0.67)**	**2.72 (0.51)**	**2.63 (0.76)**	2.93 (0.57)	**2.57 (0.63)**	**2.45 (0.50)**	**2.51 (0.71)**
n = 100							
*t*-test (F; *p*)	0.467; **0.000**	2.695; **0.002**	0.006; **0.000**	2.649; 0.081	0.945; **0.000**	5.494; **0.006**	0.039; **0.000**
Effect Size (95% CI)	1.28 (0.95, 1.64)	0.49 (0.18, 0.81)	1.29 (0.96, 1.65)	0.28 (−0.03, 0.60)	0.75 (0.44, 1.08)	0.48 (0.17, 0.80)	0.91 (0.59, 1.25)
**Perceived health status**							
Very bad, bad, fair							
Mean (SD)	**1.86 (0.75)**	2.52 (0.57)	**1.95 (0.85)**	**2.73 (0.69)**	**2.11 (0.70)**	**2.12 (0.55)**	**1.99 (0.67)**
n = 65							
Good, very good							
Mean (SD)	**2.32 (0.78)**	2.67 (0.55)	**2.42 (0.89)**	**2.94 (0.59)**	**2.55 (0.63)**	**2.48 (0.59)**	**2.41 (0.81)**
n = 101							
*t*-test (F; *p*)	0.063; **0.000**	0.042; 0.096	0.355; **0.001**	2.471; **0.042**	1.365; **0.000**	0.468; **0.000**	6.093; **0.000**
Effect Size (95% CI)	0.60 (0.28, 0.92)	0.27 (−0.04, 0.59)	0.53 (0.22, 0.86)	0.33 (0.02, 0.65)	0.67 (0.35, 1.00)	0.62 (0.31, 0.95)	0.55 (0.24, 0.88)
**Birth country**							
Spain							
Mean (SD)	**2.10 (0.81)**	2.61 (0.58)	2.20 (0.92)	2.85 (0.66)	2.36 (0.71)	2.33 (0.61)	2.23 (0.80)
n = 151							
Foreign country							
Mean (SD)	**2.56 (0.56)**	2.69 (0.29)	2.55 (0.65)	2.93 (0.33)	2.55 (0.49)	2.44 (0.43)	2.43 (0.59)
n = 15							
*t*-test (F; *p*)	5.722; **0.009**	4.038; 0.335	5.731; 0.073	4.384; 0.409	5.397; 0.191	1.135; 0.482	2.110; 0.330
Effect Size (95% CI)	0.58 (0.05, 1.12)	0.14 (−0.39, 0.68)	0.39 (−0.14, 0.93)	0.12 (−0.41, 0.66)	0.27 (−0.26, 0.81)	0.18 (−0.35, 0.72)	0.25 (−0.28, 0.79)
**Marital status**							
Single, separated, widowed							
Mean (SD)	2.07 (0.82)	2.55 (0.62)	2.20 (0.94)	**2.75 (0.67)**	2.27 (0.71)	**2.22 (0.58)**	2.21 (0.85)
n = 78							
Married							
Mean (SD)	2.21 (0.79)	2.68 (0.51)	2.27 (0.87)	**2.95 (0.59)**	2.47 (0.67)	**2.45 (0.60)**	2.27 (0.73)
n = 88							
*t*-test (F; *p*)	0.550; 0.271	2.383; 0.138	1.565; 0.658	0.513; **0.043**	0.098; 0.056	0.178; **0.013**	2.309; 0.624
Effect Size (95% CI)	0.17 (−0.13, 0.48)	0.23 (−0.08, 0.54)	0.08 (−0.23, 0.39)	0.32 (0.01, 0.63)	0.29 (−0.02, 0.60)	0.39 (0.08, 0.70)	0.08 (−0.23, 0.38)

eHealth Literacy Questionnaire (eHLQ), standard deviation (SD), and confidence interval (CI). Significant differences are printed in bold (*p* < 0.05); effect size calculated using Cohen’s d. Interpretation of ES: “small” > 0.20–0.50 SD, “medium” 0.50–0.80 SD, and “large” > 0.80 SD.

**Table 2:** Multiple regression model for eHealth literacy.

Predictors		Beta	*p* Value
**Dimension 1** **Using technology to process** **health information** R 0.641/R^2^ 0.410/adjusted R^2^ 0.396/F 10.674			
	Constant	2.244 (2.015, 2.474)	0.000
	Age group: <65 years	0.585 (0.348, 0.821)	0.000
	Secondary education: incomplete	−0.496 (−0.736, −0.255)	0.000
Marital status: single/separated/widower		−0.217 (−0.411, −0.022)	0.029
Perceived health status: very bad/bad/fair		−0.213 (−0.416, −0.011)	0.039
**Dimension 2** **Understanding of health concepts** **and language** R 0.233/R^2^ 0.054/adjusted R^2^ 0.048/F 9.303			
	Constant	2.719 (2.609, 2.829)	0.000
	Secondary education: incomplete	−0.268 (−0.441, −0.094)	0.003
**Dimension 3** **Ability to actively engage** **with digital services** R 0.647/R^2^ 0.419/adjusted R^2^ 0.411/F 57.948			
	Constant	2.074 (1.843, 2.305)	0.000
	Age group: <65 years	0.783 (0.523, 1.042)	0.000
	Secondary education: incomplete	−0.538 (−0.802, −0.273)	0.000
	**Dimension 4** **Feel safe and in control** R 0.257/R^2^ 0.066/adjusted R^2^ 0.055/F 5.702		
	Constant	2.851 (2.705, 2.996)	0.000
	Occupation: employed	0.278 (0.082, 0.475)	0.006
	Marital status: single/separated/widower	−0.205 (−0.396, −0.015)	0.035
	**Dimension 5** **Motivated to engage with** **digital services** R 0.448/R^2^ 0.201/adjusted R^2^ 0.186/F 13.400		
	Constant	2.776 (2.609, 2.942)	0.000
	Secondary education: incomplete	−0.427 (−0.629, −0.226)	0.000
	Perceived health status: very bad/bad/fair	−0.333 (−0.535, −0.130)	0.001
	Marital status: single/separated/widower	−0.207 (−0.399, −0.014)	0.036
	**Dimension 6** **Access to digital services that work** R 0.395/R^2^ 0.156/adjusted R^2^ 0.140/F 9.867		
	Constant	2.464 (2.302, 2.626)	0.000
	Age group: <65 years	0.239 (0.063, 0.415)	0.008
	Perceived health status: very bad/bad/fair	−0.307 (−0.486, −0.128)	0.001
	Marital status: single/separated/widower	−0.264 (−0.437, −0.091)	0.003
	**Dimension 7** **Digital services that suit** **individual needs** R 0.529/R^2^ 0.280/adjusted R^2^ 0.262/F 15.485		
	Constant	2.125 (1.859, 2.390)	0.000
	Age group: <65 years	0.482 (0.228, 0.735)	0.000
	Sex: Male	0.233 (0.023, 0.443)	0.030
	Secondary education: incomplete	−0.312 (−0.573, −0.050)	0.020
	Perceived health status: very bad/bad/fair	−0.228 (−0.447, −0.009)	0.041

## Data Availability

The data presented in this study are available on request from the corresponding author.
